# Liver stiffness measured by two-dimensional shear-wave elastography predicts hepatic vein pressure gradient at high values in liver transplant candidates with advanced liver cirrhosis

**DOI:** 10.1371/journal.pone.0244934

**Published:** 2021-01-07

**Authors:** Sona Frankova, Mariia Lunova, Halima Gottfriedova, Renata Senkerikova, Magdalena Neroldova, Jozef Kovac, Eva Kieslichova, Vera Lanska, Petr Urbanek, Julius Spicak, Milan Jirsa, Jan Sperl

**Affiliations:** 1 Department of Hepatogastroenterology, Transplant Centre, Institute for Clinical and Experimental Medicine, Prague, Czech Republic; 2 Laboratory of Experimental Hepatology, Institute for Clinical and Experimental Medicine, Prague, Czech Republic; 3 Charles University, First Faculty of Medicine, Prague, Czech Republic; 4 Department of Diagnostic and Interventional Radiology, Institute for Clinical and Experimental Medicine, Prague, Czech Republic; 5 Anaesthesiology, Resuscitation and Intensive Care Department, Institute for Clinical and Experimental Medicine, Prague, Czech Republic; 6 Department of Biostatistics, Institute for Clinical and Experimental Medicine, Prague, Czech Republic; 7 Department of Internal Medicine, Central Military Hospital, Prague, Czech Republic; Medizinische Fakultat der RWTH Aachen, GERMANY

## Abstract

Liver stiffness is a reliable non-invasive predictor of Hepatic Venous Pressure Gradient (HVPG) above 10 mm Hg. However, it failed to predict higher thresholds of HVPG. Our aim was to investigate whether liver stiffness and selected previously published non-invasive blood biomarkers could predict higher HVPG thresholds in liver transplant candidates without ongoing alcohol use. One hundred and nine liver transplant candidates with liver cirrhosis of various aetiologies underwent direct HVPG measurement, liver stiffness measurement by 2D shear-wave elastography (Aixplorer Multiwave, Supersonic Imagine, France) and assessment of blood HVPG biomarkers (osteopontin, VCAM-1, IL-6, TNF-α, IL-1ra/IL-1F3 and ELF score). The correlation between liver stiffness and HVPG was linear up to 30 mm Hg of HVPG (r = 0.765, p < 0.0001). The regression lines had similar slopes for HVPG values below and above 16 mm Hg (p > 0.05) and the correlation in patients with HVPG <16 mm Hg (r = 0.456, p = 0.01) was similar to patients with HVPG ≥ 16 mm Hg (r = 0.499, p < 0.0001). The correlation was similar in the subgroup patients with alcoholic (r = 0.718, p < 0.0001), NASH (r = 0.740, p = 0.008), cryptogenic (r = 0.648, p = 0,0377), cholestatic and autoimmune (r = 0.706, p < 0.0001) and viral cirrhosis (r = 0.756, p < 0.0001). Liver stiffness distinguished patients with HVPG above 16, and 20 mm Hg with AUROCs 0.90243, and 0.86824, sensitivity 0.7656, and 0.7027, and specificity 0.9333, and 0.8750. All studied blood biomarkers correlated better with liver stiffness than with HVPG and their AUROCs did not exceed 0.8 at both HVPG thresholds. Therefore, a composite predictor superior to liver stiffness could not be established. We conclude that liver stiffness is a clinically reliable predictor of higher HVPG thresholds in non-drinking subjects with advanced liver cirrhosis.

## Introduction

Portal hypertension (PH) is the main complication of liver cirrhosis contributing to the development of its life-threatening complications. Hepatic venous pressure gradient (HVPG) represents the reference standard for evaluation of the presence and severity of PH in patients with cirrhosis [1]. HVPG is presumably the most often validated tool for assessing prognosis in patients with liver cirrhosis. HVPG higher than 10 mm Hg is considered clinically significant portal hypertension (CSPH) [2]. Patients with CSPH are at risk of oesophageal varices [3–5], develop ascites and cirrhosis decompensation [6]. HVPG higher than 12 mm Hg is associated with the risk of variceal bleeding, HVPG more than 16 mm Hg with high mortality [7] and HVPG above 20 mm Hg predicts failure to control variceal bleeding [8]. HVPG measurement by hepatic vein catheterization is an invasive procedure and therefore, there is a need for an easy and accurate non-invasive method.

The use of liver stiffness (LS) as a non-invasive predictor of PH has been extensively studied in the last decade. LS measurement by transient elastography showed good predictive value in the diagnosis of both HVPG ≥ 10 mm Hg and gastroesophageal varices, and platelet count or spleen diameter were identified as parameters improving the accuracy of the prediction. Platelet count was included in the Baveno VI statement saying that upper gastrointestinal endoscopy could be avoided in patients with compensated cirrhosis and LS < 20 kPa together with platelet count >150 x 10^9^/L [9–11]. Later on, two-dimensional real time shear-wave elastography (2D-SWE) has been introduced into clinical practice [12, 13] allowing both LS and spleen stiffness measurement even in patients with ascites. Two sequential algorithms based on LS followed by spleen stiffness measurements using 2D-SWE with excellent diagnostic accuracy for CSPH have recently been proposed by Jansen [14, 15] and validated by Elkrief [16, 17]. In the meantime, Buck et al. [18] described a good correlation between blood inflammatory biomarkers and HVPG and proposed a composite diagnostic test based on four biomarkers, which was able to identify 86% of compensated cirrhotic patients with HVPG below 12 mm Hg. Some fibrogenesis biomarkers also showed correlation with HVPG but their predictive value has not been studied [19].

As mentioned above, the researchers focused mainly on the non-invasive prediction of HVPG ≥ 10 mm Hg. There are limited data on the non-invasive prediction of HVPG at levels higher than 10 mm Hg; Kim et al. showed a good ability of LS by 2D-SWE to predict HVPG ≥ 12 mm [20]. The non-invasive prediction of HVPG ≥ 16 mm Hg was described only in the study by Gouya et al. The authors evaluated portal, azygos vein and aortal blood flow by phase contrast MRI and demonstrated that azygos flow was a good predictor of HVPG ≥ 16 mm Hg [21]. Loss of correlation or weak correlation [22] between LS and HVPG above 10 mm Hg was the reason impeding prediction of higher levels of PH in most published studies. The weak correlation can be explained by the fact that increase of LS reflects predominantly increased intrahepatic resistance due to liver fibrosis and sinusoidal dysfunction whereas hyperdynamic circulation aggravates portal hypertension only at high pressure values [23]. The mentioned mechanism is supported by Reiberger et al. who demonstrated that treatment with non-selective β-blockers (NSBB) improved the correlation between LS and HVPG in patients with HVPG > 12 mm Hg [24]. NSBB probably ameliorate both hyperdynamic circulation and sinusoidal dysfunction [25]. Another factor contributing to weaker correlation between LS and HVPG may be liver steatosis since LS becomes overestimated in patients with steatosis whereas HVPG pressure value is not affected [26, 27].

Advanced NASH liver cirrhosis is generally associated with decline of steatosis regardless of high BMI [28, 29]. Contrarily, active alcohol drinkers with advanced liver cirrhosis may have severe steatosis [30]. Therefore, we assume that a different proportion of active alcohol drinkers with various degree of liver steatosis may negatively impact the correlation between LS and HVPG. Liver steatosis is also regularly associated with genotype 3 infection in patients with HCV cirrhosis. This should be considered when explaining the weaker correlation between LS and HVPG above 10 mm Hg in the study by Vizzutti et al. [31].

In this study we focused on non-invasive predictors of HVPG in a group of liver transplant candidates with advanced liver cirrhosis. These candidates represent a unique group of patients with advanced liver cirrhosis characterized by almost complete absence of active alcohol abusers. Since vast majority of such patients have CSPH, these patients need an accurate assessment of potential risk of complications, knowledge of which is the prerequisite for correct treatment during the waiting period. Moreover, despite the fact that NSBB are no more recommended in majority of liver transplant candidates, the rest may still benefit from NSBB administration [32]. Therefore, a non-invasive method for estimation of HVPG in liver transplant candidates capable to evaluate the response to NSBB would be beneficial. Our aim was to assess the predictive power for HVPG of LS and selected blood biomarkers of inflammation and fibrogenesis and their combination(s) in a carefully selected cohort of these steatosis-free patients.

### Patients and study design

This prospective, monocentric study included liver transplant candidates with liver cirrhosis admitted between October 2016 and July 2018 to The Department of Hepatogastroenterology at the Institute for Clinical and Experimental Medicine, Prague, Czech Republic. All the patients were admitted to the hospital for evaluation before enrolment into waiting list and followed a protocol-defined work-up, including LS and HVPG measurement. A total number of 119 consecutive patients were eventually prospectively enrolled in the study, ten patients were subsequently excluded from further evaluation. Their demographic data are summarized in Tables 1 and 2. All patients were abstaining from alcohol for at least six months. Exclusion criteria were: complete or partial thrombosis of the portal vein or its right branch diagnosed by abdominal ultrasound or computed tomography, history of transjugular intrahepatic portosystemic shunt (TIPS), hepatorenal syndrome requiring vasoactive drug administration or renal replacement therapy, pulmonary hypertension diagnosed by echocardiography, severe bacterial infection or sepsis, variceal bleeding occurring within 4 weeks prior to hepatic vein catheterisation, and hepatocellular carcinoma outside of the Milan criteria [33]. The patients were screened to assess inclusion and exclusion criteria (clinical examination, routine blood tests, abdominal ultrasound, abdominal CT scan, gastroscopy and LS measurement). The day following LS measurement, they underwent HVPG measurement by liver vein catheterisation and blood sampling for plasma markers of portal hypertension. The study was approved by local Institutional Review Board (IRB of Institute for Clinical and Experimental Medicine and Thomayer’s Hospital, Prague). All patients signed the informed consent with the study. The patients were selected for enrolment into the waiting list according to the published medical criteria after the evaluation process [34]. Organ donation was driven by the Czech law No. 285/2002 Coll., On donation, grafts and transplantation of tissues which is compatible with the European Union legislative. The Czech law applies the principle of presumed consent, i.e. the informed consent with organ or tissue donation is not required. People who, during their lifetime, express a clear opposition to the donation in writing and are registered in the National Registry of people opposed to the post-mortem withdrawal of tissues and organs are excluded from the organ donation. All the donors were referred to our transplant centre from regional hospitals across the Czech Republic via Coordination Centre of Transplantations (https://kst.cz/en/) which is a state organization controlled by the Ministry of Health of the Czech Republic. The organ removal can be performed only after consent of the Coordination Centre of Transplantations that administrates the waiting list and National Registry of people opposed to the post-mortem withdrawal of tissues and organs. The Centre manages organs allocation among the transplant centres.

**Table 1:** Baseline clinical and laboratory characteristics of the whole cohort and patient subgroups.

Variable [median, range]	All patients N = 109 (100%)	Child-Pugh A N = 30 (27.5%)	Child-Pugh B/C N = 79 (72.5%)	p
Age [years]	61 (21–84)	66 (38–84)	58 (21–73)	< 0.001
Gender [Male]	73 (67.0%)	23 (76.7%)	50 (63.3%)	N.S.
BMI [kg/m^2^]	26.7 (18.4–46.8)	28.6 (20.4–46.8)	25.5 (18.4–38.5)	0.006
Child-Pugh score [points]	8 (5–13)	5 (5–6)	8 (7–13)	< 0.001
Aetiology of liver cirrhosis				< 0.001
Alcohol	38 (34.9%)	10 (33.3%)	28 (35.5%)	
NASH	11 (10.1%)	5 (16.7%)	6 (7.6%)	
Cryptogenic	10 (9.2%)	1 (3.3%)	9 (11.4%)	
Viral	23 (21.1%)	14 (46.7%)	9 (11.4%)	
(HBV/HCV)	(5/18)	(3/11)	(2/7)	
Cholestatic and autoimmune	25 (22.9%)	0 (0%)	25 (31.6%)	
Metabolic—Wilson disease	2 (1.8%)	0 (0%)	2 (2.5%)	
HCC within Milan criteria	38 (34.9%)	20 (66.7%)	18 (22.8%)	< 0.001
MELD score [points]	14 (6–37)	9 (6–21)	15 (7–37)	< 0.001
Overt hepatic encephalopathy	13 (11.9%)	0 (0%)	13 (16.5%)	0.02
Spleen diameter [cm]	15 (9–25)	14 (9–18)	16 (9–25)	0.004
Oesophageal varices (none/small/large)	33/32/44 (30.3/29.4/40.3%)	13/ 7/10 (43.7/23.3/33.3%)	20/25/34 (25.3/31.6/43.1%)	N.S.
History of variceal bleeding	19 (17.5%)	4 (13.3%)	15 (19.0%)	N.S.
Ascites (none/small/large)	55/25/29 (50.4/23.0/26.6%)	27/3/0 (90.0/10.0/0.0%)	28/22/29 (35.4/27.8/36.8%)	< 0.001
Platelets count [x10^9^/L]	98 (40–344)	102 (48–286)	98 (40–344)	N.S.
Bilirubin [μmol/L]	34 (5–257)	20 (5–31)	47 (10–257)	< 0.001
Albumin [g/L]	30 (17–49)	39 (29–49)	28 (17–43)	< 0.001

**Table 2:** Liver stiffness, HVPG and blood predictors of HVPG and fibrosis.

Variable [median, range]	All patients N = 109 (100%)	Child-Pugh A N = 30 (27.5%)	Child-Pugh B/C N = 79 (72.5%)	p
Liver stiffness median [kPa]	29.7 (9.2–61.6)	19.3 (10.7–39.8)	33.6 (9.2–61.6)	< 0.001
HVPG median [mm Hg]	17 (5–31)	13 (5–20)	18 (6–31)	< 0.001
LSPS [points]	4.4 (0.6–27.9)*	3.0 (0.8–8.8)^+^	5.4 (0.6–27.9)^a^	< 0.001
ELF score	12.5 (8.7–16.4)	11.7 (8.7–14.0)	12.7 (10.1–16.4)	< 0.001
Osteopontin [ng/mL]	142 (52–439)**	93 (54–169)	172 (52–439)^aa^	< 0.001
VCAM-1 [ng/mL]	2718 (579–10268)**	1635 (579–4509)	3348 (1134–10268)	< 0.001
TIMP-1 [ng/mL]	462 (180–1834)	313 (180–562)	515 (226–1834)	< 0.001
PIIINP [ng/mL]	21.8 (6.3–87.7)	16.7 (7.9–50.2)	27.5 (6.3–87.7)	< 0.001
TNF-α [pg/mL]	2.3 (0.1–6.5)***	2.1 (0.1–3.8)^++^	2.4 (0.3–6.5)^aaa^	0.02
IL1-Ra/IL-1-F3 [pg/mL]	265 (65–1689) **	331 (171–728)^++^	254 (65–1689)	N.S.
Hyaluronic acid [ng/mL]	560 (20–20639)	311 (20–1356)	662 (57–20639)	< 0.001

*n = 104,

**n = 107,

***n = 105,

^+^n = 26,

^++^n = 29,

^a^n = 78,

^aa^n = 77,

^aaa^n = 76

### Blood sampling

The study subjects were in a sitting position for at least 5 min (but not >10 min) before and during sampling. Venous blood was taken between 8 and 10 a. m. The Vacuette system (VACUETTE^®^ TUBE 8 mL Z Serum Separator Clot Activator cat. No. 455071, and VACUETTE^®^ 9 ml K3 EDTA Plasma Separator cat. No. 455036, both from Greiner Bio-One, Kremsmünster, Austria) was used together with 21-gauge needles (Greiner Bio-One). Separation of blood corpuscles was done within 60 min. after sampling at 3000 g for 10 min. (centrifuge Beckman Allegra, Beckman Coulter, Indianapolis, IN). Several serum and plasma aliquots of 500 μL were prepared within 60 min. after centrifugation. CryoKing tubes from Biologix Group Limited, Jinan, China, cat. No. 89–3101, were used to store serum and plasma aliquots at −80°C until analysis.

### Analytical methods

Serum concentrations of hyaluronic acid (HA), Amino-Terminal Propeptide of Type III Procollagen (PIIINP), and Tissue Inhibitor of Matrix Metalloproteinase 1 (TIMP-1) were measured by the ADVIA Centaur^®^ HA assay, lot 25,215,019, the ADVIA Centaur^®^ PIIINP assay, lot 26,290,023, and the ADVIA Centaur^®^ TIMP-1 assay, lot 28,900,016, respectively (Siemens Healthineers, Erlangen, Germany). ADVIA Centaur ELF calibrator was used for calibration of HA, PIIINP, and TIMP-1 assays and ADVIA Centaur ELF quality control materials (three levels) were used as assay controls. Repeatability (within-run CV) assessed as declared by the manufacturer was < 5.6, < 4.2, and < 3.3% for HA, PIIINP and TIMP-1, respectively. Intermediate precisions (between-run CVs) were < 3.2, < 5.1, and < 5.5%. The respective measurement ranges for HA, PIIINP, and TIMP-1 were 1.6–1000, 0.5–150, and 3.5–1300 ng/mL. Traceability was not provided by the manufacturer. Limits of detection of HA, PIIINP, and TIMP-1 were 1.6, 0.5 and 3.5 ng/mL. All measurements were performed in one run during one day by the same laboratory technician using a Centaur CP immunochemistry analyzer (Siemens Healthineers). The Enhanced Liver Fibrosis (ELF) was calculated according to the Centaur CP formula: 0.846 x ln (HA) + 0.735 x ln (PIIINP) + 0.391 x ln (TIMP-1) + 2.494.

Interleukin-6 (IL-6), Vascular Cell Adhesion Molecule 1 (VCAM-1), Interleukin-1 Receptor Antagonist (IL-1ra/IL-1F3), Osteopontin and Tumour Necrosis Factor alpha (TNFα) were assessed in plasma samples obtained from the study subjects according to the manufacturer’s instructions using the assays No. HS600B, DVC00, DRA00B, DOST00 and HSTA00E from R&D Systems, Minneapolis, MN. The absorbance was measured on a Synergy^™^ 2 Multi-Detection Microplate Reader (BioTek Instruments, Winooski, VT).

### HVPG measurement

Catheterization of the hepatic vein was performed to measure HVPG. Using the transjugular route, an open-end zero-side holes 5F multipurpose angiographic catheter (Cordis, Santa Clara, CA), was inserted through a 6F sheath (Super Arrow-Flex Percutaneous Sheath Introducer Set, Arrow International brand of Teleflex, Wayne, PA). Iodinated radiological contrast medium was injected into the right or middle hepatic vein to confirm the position of the catheter in a wedged position by fluoroscopy. The pressure was measured five times to demonstrate reproducibility, the mean value was then used for further calculations. HVPG was calculated as the difference between wedged and free hepatic venous pressures. The radiologist performing catheterization was blinded to elastography measurement results.

### Liver stiffness measurement

After at least 10 hours of fasting, 2D-SWE was performed using the Aixplorer^®^ ultrasound system (Supersonic Imagine S.A., Aix-en-Provence, France) with an abdominal 3.5 MHz curved array probe (SC6-1) as recommended by three experienced radiologists (more than 50 exams each). 2D-SWE measurements were performed within 7 days before or after HVPG measurement.

The operator was not aware of HVPG results when performing 2D-SWE. LS measurements were performed on the right lobe of the liver through the intercostal spaces with the patient in the supine position and the right arm maximally abducted. All RT-SWE acquisitions were performed using a 3.5 x 2.5 cm box, placed at more than 2 cm under the liver capsule, avoiding large vessels. During the examination the patient was requested to hold breath as needed. After obtaining a stable and homogenous elastographic image inside the box, a region of interest (ROI) was selected using the Q-box tool and placed in the most homogeneous area and the median values of LS within the ROI was displayed and registered. The diameter of the Q-box was set > 15 mm. Three elastographic images from different liver areas were obtained in all patients and the mean value was used for further calculations.

### Other known or potential non-invasive predictors of portal hypertension

The MELD score (Model for End-Stage Liver Disease) is a composite predictor of survival in patients with cirrhosis calculated from total serum bilirubin, serum creatinine and the international normalized ratio (INR). MELD score was originally invented to predict short-term survival in cirrhotic patients [35]. Later studies showed that MELD score correlates with MR elastography results, presence of varices and mortality in patients with variceal bleeding [36, 37] and is currently used as a tool for donor livers allocation [35, 38, 39].

LSPS (Liver Spleen Platelets Score) is also a composite predictor combining LS, platelets count and spleen diameter [40]. LSPS was calculated as described previously: [LS (in kiloPascals) x spleen diameter (in centimetres)]/platelet count ratio (x10^9^/L). LSPS was superior to LS alone for identification of patients with CSPH in a study by Berzigotti [40].

IL-6, IL1-Ra/IL-1-F3, and VCAM-1 are inflammatory biomarkers correlating most significantly with HVPG in the study [18]. TNFα being also an inflammatory biomarker correlated with prehepatic portal hypertension in animal experiments [41, 42]. Osteopontin, acting as a key component of bone matrix and multifunctional cytokine, also correlated well with HVPG in humans [19].

The ELF score was shown to correlate with the stage of liver fibrosis in liver diseases of various aetiologies [43–45]. Therefore, we decided to evaluate also the score and its individual components as potential markers of portal hypertension.

### Statistical analysis

The statistical analysis was performed using the SigmaPlot 11.0 (Systat Software Inc., San Jose, CA) or JMP 11.0.0 (2013, SAS Institute Inc., Cary, NC). Quantitative data are presented as median and range and qualitative data are reported as percentage (%). Shapiro-Wilk test was used to evaluate the normal distribution of data. Spearman’s test was used for correlations among continuous variables. Medians were compared using t-test or Mann-Whitney test, as appropriate. The diagnostic performance of each non-invasive parameter was assessed by receiver operating characteristic (ROC) curves analysis. Optimal cut-off values were calculated using a common optimization step that maximized the Youden index. The performance of tested non-invasive parameters to predict various levels of portal hypertension was estimated by calculating the proportion of correctly classified patients—diagnostic accuracy, together with the sensitivity, specificity, positive predictive value (PPV), negative predictive value (NPV), and likelihood ratios (LR). The Fischer’s exact test and McNemar’s test were used in the 2 × 2 contingency tables for assessing differences in the proportion of misclassified patients with dichotomous cut-offs, as well as for comparing categorical variables. For all calculations, a p value < 0.05 was considered to indicate statistical significance. A pre-study statistical sample size assessment was conducted based on results obtained in patients assessed by the same method for LS and HVPG during one year before the study onset; to achieve a correlation r = 0.699 with a power of 0.9, for a level of significance α = 0.05, at least 24 patients should have been enrolled.

## Results

### Patient characteristics

A total number of 119 consecutive patients with advanced liver cirrhosis of various aetiology admitted as liver transplant candidates were prospectively enrolled in the study. Ten patients were subsequently excluded from further evaluation: 7 patients for inability to perform representative LS measurement in 3 ROIs, 2 patients for pulmonary hypertension diagnosed by echocardiography and 1 patient who owned up to excessive alcohol consumption. Interestingly, none of the 7 patients in whom the LS measurement failed had BMI higher than 30. The final number of patients who underwent both LS and HVPG measurement and thus could be evaluated was 109. Eighty-three patients underwent liver transplantation at the time of the manuscript preparation. Liver cirrhosis was proved in all of them and none of them had steatosis in > 5% of hepatocytes in the explanted liver. The median period between study recruitment and liver transplantation was 109 days (range 10–769 days). All the patients received grafts from the heart-beating donors after brain death, age of the donors ranged from 21 to 81 years. None of the organ donors was from a vulnerable population and none of them was registred in the National Registry of people opposed to the post-mortem withdrawal of tissues and organs. For further evaluation, the patients were divided into two groups according to the stage of liver disease based on the Child-Pugh classification: group CPS-A including patients with Child-Pugh class A (30/109, 27.5%) and group CPS-B/C including patients with Child-Pugh class B or C (79/109, 72.5%). The obtained HVPG values ranged from 5 to 31 mm Hg. Only 12/109 (11.0%) patients had HVPG lower than 10 mm Hg; the remaining 97/109 (91%) patients had CSPH. A detailed characterisation of the patient cohort is presented in Tables 1 and 2, the complete patients’ characteristics are included in the S1 File.

**Fig 1:**
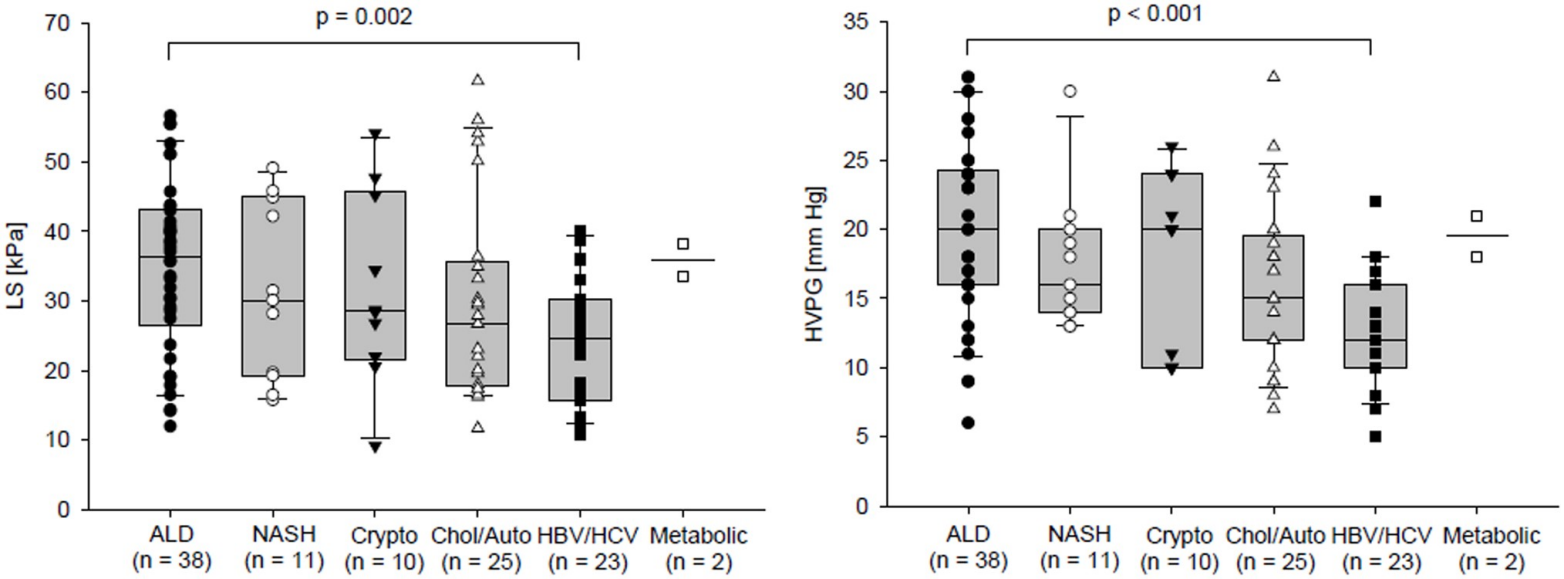
Comparison of LS and HVPG between groups of patients according to the aetiology of the liver cirrhosis. The data of two patients with metabolic liver disease were displayed but not included in the statistical analysis.

HPVG and LS values were also compared among the groups classified according to the aetiology of liver cirrhosis. Comparisons were done using the All Pairwise Multiple Comparison Procedures (Holm-Sidak method) at overall significance level 0.05. HVPG was significantly higher in patients with alcoholic liver cirrhosis compared to patients with liver cirrhosis of viral aetiology (19.97±6,58 vs. 12.78±4.24 mm Hg, p <0.001). Similarly, the same groups showed significant difference in LS value; LS was also higher in patients with alcoholic liver cirrhosis compared to those with liver cirrhosis of viral aetiology (34.93±12.37 vs. 24.49±9.32 kPa, p = 0.005) (Fig 1).

### Relations between the studied biomarkers

**Fig 2:**
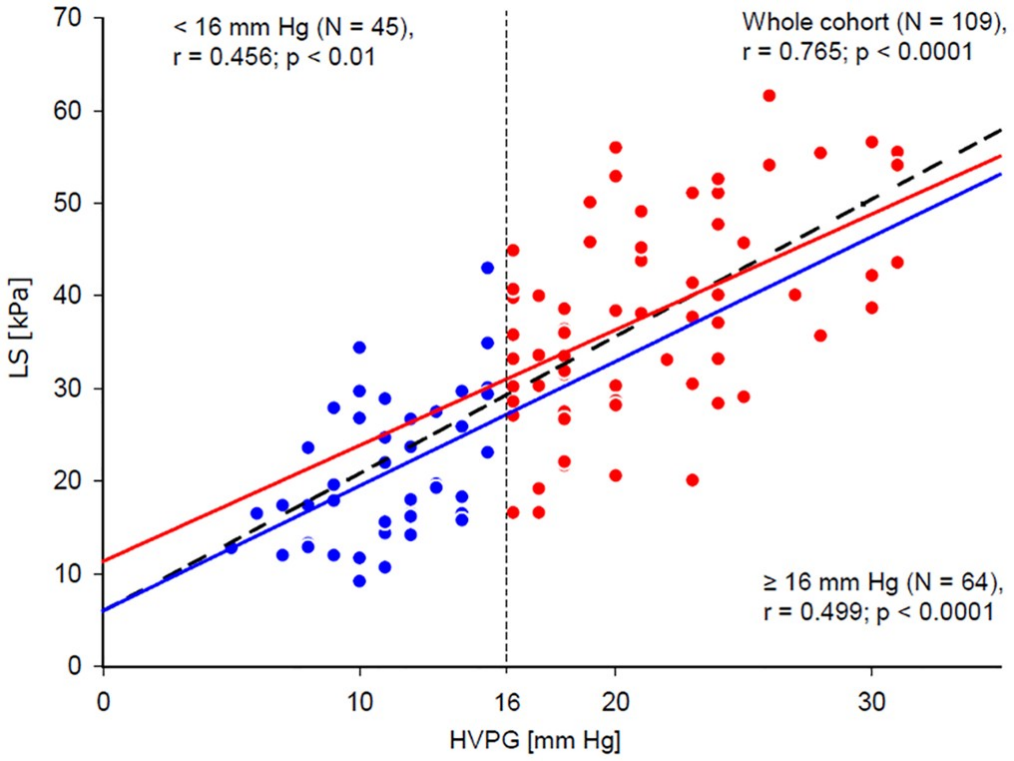
Relation between LS and HVPG. Blue line represents linear fit calculated for patients with HVPG lower than 16 mm Hg (represented by blue dots), red line shows the same for patients having HVPG equal to or higher than 16 mm Hg (represented by red dots). Dashed black line shows linear fit calculated for all patients together. **All patients:** β = 1.52, 95%CI = (1.27–1.77), p <0.001; LS [kPa] = 4.9 + 1.5 x HVPG [mmHg]. **<16 mm Hg:** β = 1.34, 95%CI = (0.59–2.10), p = 0,001; LS [kPa] = 6.0 + 1.3 x HVPG [mmHg]. **≥16 mm Hg:** β = 1.25, 95%CI = (0.73–1.77), p <0.001; LS [kPa] = 11.4 + 1.2 x HVPG [mmHg].

**Fig 3:**
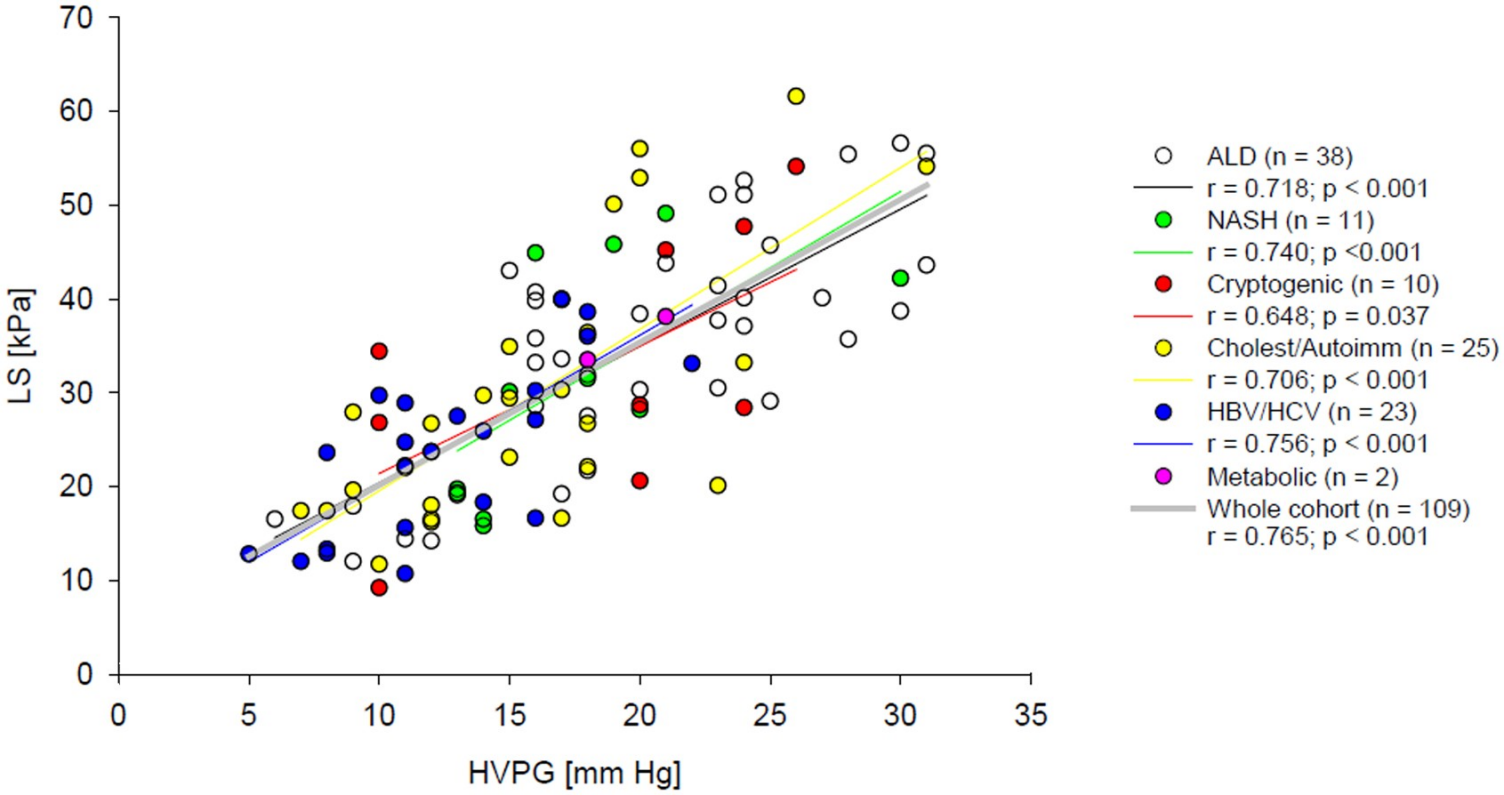
Relation between LS and HVPG. Coloured lines represent linear fits calculated for patients with alcoholic, NASH, cryptogenic, cholestatic/ autoimmune and viral (HBV and HCV) cirrhosis, dotted black line shows linear fit calculated for all patients together.

As a first step of the data analysis, correlations between HVPG, LS and non-invasive blood predictors of portal hypertension were calculated. The obtained Spearman’s non-parametric correlation coefficients are presented in Table 3. The strongest correlation with HVPG was achieved for LS. Osteopontin level was the best blood marker for both HVPG and LS and its correlation coefficients with HVPG and LS were closely similar. The remaining blood predictors of portal hypertension correlated better with LS than with HVPG; however, their correlations with both HVPG and LS were weaker than those of osteopontin. Correlations of ELF score and LSPS with HVPG were also calculated for CPS A and CPS B/C patient groups. ELF score did not correlate with HVPG in any of the groups whereas LSPS correlation with HVPG was stronger in the CPS-A group (r = 0.528, p = 0.006 (n = 26), than in the CPS-B/C group (r = 0.308, p = 0.006, n = 78). Importantly, the residual plot and normal distribution of residuals justified the assumed linear relationship between HVPG and LS in the whole range of HVPG values (Fig 2). Moreover, the regression lines had similar β values for patients with HVPG values below and above 16 mm Hg (p > 0.05). Aetiology of the cirrhosis had no impact on the relation between LS and HVPG as can be seen from Fig 3; linear regression was calculated separately for each of the five aetiology groups, two subjects representing the metabolic aetiology group were not included in the statistical analysis. The use of linear regression was justified by residual plots and the β values did not differ from the β value calculated for the whole cohort (Table 4).

**Table 3:** Spearman’s non-parametric correlation coefficients between HVPG, LS and the evaluated non-invasive markers.

Variable	HVPG		Liver stiffness	
	Spearman’s r	p	Spearman’s r	p
Liver stiffness [kPa]	0.765	< 0.0001	NA	N.A.
LSPS [points]	0.4488	< 0.0001	0.5046	< 0.0001
MELD score [points]	0.2990	0.0016	0.4254	< 0.0001
Spleen diameter [cm]	0.1100	N.S.	0.0627	N.S.
Platelets [x10^9^/L]	–0.0223	N.S.	0.0793	N.S.
Osteopontin [ng/mL]	0.5143	< 0.0001	0.5086	< 0.0001
VCAM-1 [ng/mL]	0.4194	< 0.0001	0.4635	< 0.0001
TIMP-1 [ng/mL]	0.4339	< 0.0001	0.5209	< 0.0001
ELF score [points]	0.3485	0.0002	0.3916	< 0.0001
HA [ng/mL]	0.2880	0.0024	0.348	0.0002
PIIINP [ng/mL]	0.2712	0.0044	0.2940	0.0019
IL-6 [pg/mL]	0.2512	0.015	0.342	0.0008
IL1-ra/IL-1F3 [pg/mL]	–0.1825	N.S.	–0.0457	N.S.
TNF-α [pg/mL]	0.0444	N.S.	0.178	N.S.

N.A. not applicable, N.S. not significant.

**Table 4:** LS vs HVPG relationship formulas and β values in groups classified according to the aetiology of liver cirrhosis.

Patient group	LS [kPa] relationship formula	Beta values, 95% CI and p
Alcoholic (N = 38)	5.8 + 1.5 x HVPG [mm Hg]	β = 1.46 (95% CI = 1.07–1.85, p < 0.0001)
NASH (N = 11)	2.7 + 1.6 x HVPG [mm Hg]	β = 1.63 (95% CI = 0.37–2.88, p = 0.0312)
Cryptogenic (N = 10)	7.8 + 1.4 x HVPG [mm Hg]	β = 1.36 (95% CI = 0.26–2.46, p = 0.0419)
Viral (HBV/HCV) (N = 23)	3.9 + 1.6 x HVPG [mm Hg]	β = 1.61 (95%CI = 0.97–2.25, p < 0.0001)
Cholestatic/autoimmune (N = 25)	2.3 + 1.7 x HVPG [mm Hg]	β = 1.72 (95% CI = 1.03–2.42, p < 0.0001)

### Non-invasive markers as predictors of HVPG cut-off points

In the next step, areas under the receiver operating characteristic curves (AUROCs) were calculated for all tested non-invasive HVPG predictors at three cut-off points. Whereas the AUROCs of LS to predict 16 and 20 mm Hg of HVPG were all higher than 0.8 (Fig 3), AUROCs of all other tested non-invasive markers to predict the same HVPG cut-offs did not exceeded 0.8 and therefore these markers were not considered suitable for clinical use (Table 5). AUROCs were not calculated for threshold 10 mm Hg since there were only 12/109 patients with HVPG lower that 10 mm Hg.

**Table 5:** AUROCs of liver stiffness and other non-invasive markers to predict HVPG 16, and 20 mm Hg.

**Cut-off point HVPG 16 mm Hg**			
	AUROC	95% CI	p value
Liver stiffness [median, kPa]	**0.9024**	0.8464–0.9585	< 0.0001
LSPS [points]	0.7608	0.6685–0.8531	< 0.0001
ELF score [points]	0.7116	0.6146–0.8086	0.00018
Osteopontin [ng/mL]	0.7765	0.6864–0.8667	< 0.0001
VCAM-1 [ng/mL]	0.7228	0.6228–0.8228	< 0.0001
TIMP-1 [ng/mL]	0.7358	0.6411–0.8304	< 0.0001
PIIINP [ng/mL]	0.6585	0.5572–0.7598	0.0049
TNF-α [pg/mL]	0.512	0.3981–0.6259	N.S.
IL-6 [pg/mL]	0.6326	0.5164–0.7487	0.028
IL1-Ra/IL-1-F3 [pg/mL]	0.4215	0.3122–0.5308	N.S.
Hyaluronic acid [ng/mL]	0.684	0.5810–0.7871	0.00118
**Cut-off point HVPG 20 mm Hg**			
	AUROC	95% CI	p value
Liver stiffness [median, kPa]	**0.8682**	0.8006–0.9359	< 0.0001
LSPS [points]	0.7151	0.6161–0.8141	0.0002
ELF score [points]	0.6837	0.5757–0.7918	0.002
Osteopontin [ng/mL]	0.7714	0.6828–0.8600	< 0.0001
VCAM-1 [ng/mL]	0.6979	0.5985–0.7972	0.001
TIMP-1 [ng/mL]	0.7241	0.6291–0.8191	0.0001
PIIINP [ng/mL]	0.6552	0.5384–0.7720	0.008
TNF-α [pg/mL]	0.5157	0.4007–0.6307	N.S.
IL-6 [pg/mL]	0.6488	0.5329–0.7648	0.03
IL1-Ra/IL-1-F3 [pg/mL]	0.4036	0.2932–0.5139	N.S.
Hyaluronic acid [ng/mL]	0.6411	0.5335–0.7488	0.016

### Diagnostic performance of liver stiffness as the strongest predictor of HVPG cut-off points

The best cut-off values of LS to predict 16 and 20 mm Hg HVPG were selected using the Youden Index (Table 6). The accuracy of LS to predict HVPG above 16 and 20 mm Hg was 83.5 and 81.7%, respectively (p < 0.05, McNemar’s test). The diagnostic accuracy of LS to predict 16 mm Hg of HVPG in CPS-A or CPS-B/C patients did not differ from the prediction for 20 mm Hg (see Table 6). The comparison of the diagnostic accuracy between CPS-A and CPS-B/C patients could not be calculated at HVPG 20 mm Hg due to the fact that all studied subjects in the CPS-A group had HVPG below 20 mm Hg.

**Table 6:** Diagnostic performance of liver stiffness to predict HVPG above 16, and 20 mm Hg*.

HVPG [mm Hg]	16	20
**AUROC**	**0.90243**	**0.86824**
**OR 95% CI**	**1.21484 1.1396–1.3174**	**1.157 1.101–1.231**
Cut-off [kPa]	30.2	37.1
Sensitivity	0.7656	0.7027
Specificity	0.9333	0.8750
PPV	94.23%	74.29%
NPV	73.68%	84.00%
+LR	11.47	5.62
–LR	0.25	0.34
Diagnostic accuracy	83.5% (91/109)	81.7% (89/109)
McNemar’s test	p > 0.05	

PPV, positive predictive values; NPV, negative predictive values; +LR, positive likelihood ratio;–LR, negative likelihood ratio;

* applicable only on a similarly selected group of patients

### Modelling of a composite predictive factor

HVPG as the dependent variable, and LS together with all other studied non-invasive predictors as independent variables were analysed by the multiple linear regression. The strongest association was found between HVPG and LS (p < 0.0001). A weaker association was found between HVPG and IL1-ra/IL-1F3 (p = 0.01874) but the stepwise modelling showed minimal increase in r^2^ after addition of IL1-ra/IL-1F3 to LS (0.5653 vs. 0.5348). Logistic regression using the same variables at 16 and 20 mm Hg identified LS as significant predictive factor at both cut-off points (p < 0.0001 at both 16 and 20 mm Hg (Table 7). None of the tested blood parameters presented with the ability to improve prediction of HVPG by LS. Comparison of correct prediction rates (2D-SWE diagnostic accuracies) among the patients with CPS-A and CPS-B/C at 16 mm Hg HVPG cut-off point revealed 83% (25/30) correctly predicted HVPG pressure values in the CPS-A group and 82% (65/79) correctly predicted HVPG pressure values in the CPS-B/C group.

**Table 7:** Composite non-invasive HVPG predictor modelling by linear and logistic regression.

	Stepwise multiple linear regression	Stepwise logistic regression at various cut-off points of HVPG	
Dependent variable	HVPG	16 mm Hg	20 mm Hg
Independent variables	p value	p value	p value
Liver stiffness [kPa]	**< 0.0001**	**<0.0001**	**< 0.0001**
MELD [points]	0.85768	0.66765	0.6270
Spleen diameter [cm]	0.85706	0.86437	0.8098
Platelets [x10^9^/L]	0.94557	0.89992	0.2541
Osteopontin (ng/mL)	0.1414	0.04254	0.3603
VCAM-1 [ng/mL]	0.14792	0.6366	0.3268
TIMP-1 [ng/mL]	0.96405	0.63196	0.8637
ELF score [points]	0.47159	0.06959	0.1879
Hyaluronic acid [ng/mL]	0.44322	0.05005	0.6911
PIIINP [ng/mL]	0.53925	0.11706	0.1092
IL-6 (pG/mL) [ng/mL]	0.7389	0.97873	0.5831
TNF-α (pG/mL) [pg/mL]	0.4951	0.29018	0.6445
IL1-ra/IL-1F3 [pg/mL]	**0.01874**	0.2667	0.1257

## Discussion

In our carefully selected cohort of patients awaiting liver transplantation due to advanced liver cirrhosis, we obtained a strong correlation between LS and HVPG in a wide range of values (r = 0.7645, p < 0.0001). The correlation remained linear even in the range from 16 to 30 mg Hg of HVPG, the AUROC values at 16 and 20 mm Hg of HVPG were all above 0.85 and the corresponding cut-off points of LS were 30.2, and 37.1 kPa, respectively. LS of more than 30.2 kPa had diagnostic accuracy 84% and 37.1 kPa 82% for the detection of HVPG higher than 16 and 20 mm Hg, respectively. However, the clinical applicability of these cut-off point should be limited only to the similarly selected group of patients. We did not present the diagnostic accuracy for the prediction of HVPG above 10 mm Hg because there were only 12 of 109 (11.0%) patients with HVPG lower than 10 mm Hg. These data confirm our hypothesis that non-homogeneity of the study group in previous studies regarding alcohol abuse might have been one of the reasons for the weak correlation between LS and HVPG values above 10 mm Hg [12].

Additional support for our hypothesis that active alcohol drinkers with advanced liver cirrhosis have significantly overestimated LS in comparison with abstainers came from the study by Conti at al. [27]. These authors demonstrated that LS in patients with liver cirrhosis is proportional to the grade of steatosis. Liver transplant candidates seem to be a unique group of patients for studies of the relation between LS and HVPG in patients with advanced liver cirrhosis due to absence of steatosis.

Another factor contributing to the uniformity of HVPG and LS relationship in our patients is the fact that liver transplant candidates are considered as patients with irreversible end-stage liver disease. There is no detailed definition of the point of no return at which liver cirrhosis becomes irreversible but patients behind this point have uniform clinical presentation and probably also uniform fibrosis and haemodynamic impairment. In contrast to this oversimplified view, Ferrusquía-Acosta et al. proved that aetiology of liver cirrhosis affects liver haemodynamics also in patients with severe portal hypertension [46]. The authors measured wedged hepatic vein pressure (WHVP) and portal pressure (PP) directly before and during TIPS insertion procedure in patients with NASH, alcohol-related and HCV cirrhosis. They found more often the disagreement between WHVP and PP in NASH than in HCV patients (37.5% vs. 14%) and PP was more frequently underestimated in patients with NASH (32.5% vs. 7.5%). On the other hand, the authors did not provide data on LS values and their correlation with HVPG.

In our study, aetiology of cirrhosis had no impact on the relation between LS and HVPG. Despite the fact that HVPG was significantly higher in the group of patients with alcoholic cirrhosis than in the group of patients with cirrhosis of viral aetiology, the correlation remained unchanged. Higher HVPG and LS in alcoholic liver cirrhosis mirrors the fact that these patients are usually enlisted for liver transplantation with more advanced liver disease than patients with viral aetiology.

Obese patients and patients with cirrhosis owing to NASH were also included in our study group. Severe steatosis should not be present in these patients because it usually fades out in the late stage of NASH cirrhosis, but obesity persists. Obesity itself may increase variability of LS measurement [47]. However, all the 7 patients with unreliable LS measurement excluded from further evaluation had BMI lower than 30 and reliable LS values were obtained in 29 patients with BMI above 30. Neither ascites presented an obstacle for LS measurement as was previously supported by Conti et al [27]. 2D-SWE seems to be the appropriate method for LS measurement in patients with advanced liver cirrhosis: the presence of ascites in most patients with advanced liver disease (more than one half in our cohort of patients) was the crucial reason why we had chosen 2D-SWE as the non-invasive tool for LS assessment in the study. Albeit Fibroscan^®^ advantages include short procedure time and the ability to perform the test at the bedside, it is nearly impossible to obtain the results from patients with ascites [48, 49].

Unfortunately, we were not able to improve the prediction of HVPG using a composite non-invasive predictor consisting of LS combined with one or more blood biomarkers because none of the tested blood biomarkers showed better correlation with HVPG than with LS. Consistently with this statistical concept, stepwise linear regression and logistic regression analysis failed to identify another independent non-invasive predictor being able to improve prediction of HVPG by LS. We did not measure the spleen stiffness because at the beginning of our study, the software upgrade of the 2D-SWE device for spleen stiffness measurement was not available and the measurement of spleen stiffness using liver mode was considered an off-label procedure according to the manufacturer. Furthermore, the results of spleen stiffness measured using the liver mode, which we had evaluated retrospectively before this study, were disappointing since spleen stiffness correlation with HVPG was weak (r = 0.320, p = 0.02, n = 32). Consistently with our experience, spleen stiffness measured by 2D-SWE in the study by Procopet et al. [12] had a lower AUROC for diagnosing CSPH compared with LS. Jansen et al. demonstrated later that 2D-SWE measurement of liver stiffness correlated better with HVPG than 2D-SWE spleen stiffness [14] and proposed a sequential measurement of LS and spleen stiffness for the diagnosis of CSPH. The sequential algorithm was validated by Elkrief et al. [17] 2D-SWE is not considered a suitable method for spleen stiffness measurement [23]; vibration-controlled transient elastography (VCTE, Fibroscan^®^) combined with B-mode ultrasound localisation of the spleen or point SWE seem to be more promising for CSPH diagnosis [50–52]. However, spleen stiffness measurement seems to be a suitable non-invasive tool for evaluation of response to NSBB. Two recent studies using pSWE [53]and VCTE [54] technique indicate promising results in non-invasive NSBB response evaluation but further data and standardization is needed in this field. Absence of spleen stiffness measurement could be considered a weak point of our study; nonetheless, based on the above-mentioned data, we could have not expected a breaking improvement of HVPG prediction using also spleen stiffness measurement.

Based on the results of study by Bruha and colleagues [19], we initially considered osteopontin as the most promising blood biomarker. However, Bruha et al. [19] achieved a weaker correlation (r = 0.25, p = 0.0022, vs. r = 0.5143, p < 0.0001) between HVPG and plasma osteopontin concentration in their group of 154 patients with liver cirrhosis of various aetiology. The authors declared that none of the patients was an excessive drinker; however, not all were absolutely abstaining. This was probably the reason why the AUROCs did not exceed the value of 0.8 at any point (16 and 20 mm Hg) of HVPG in their study.

LSPS predicted better CSPH than LS alone in the study reported by Berzigotti et al. [40]. LSPS is a composite predictor; it was superior in the prediction of CSPH than LS alone, but the predictive value of LSPS for higher thresholds was not investigated. The study group by Berzigotti [40] included predominantly CPS-A patients, only a few patients of CPS-B but none of CPS-C class. Not surprisingly, the correlation of LSPS with HVPG was weak in our entire group (r = 0.449, p < 0.0001) consisting predominantly of CPS-B and C patients. When evaluating the correlation between LSPS and HVPG separately in CPS-A and CPS-B/C groups, we noted that the correlation in the CPS-A group was strong despite the low number of patients in this group. In the group of CPS-B/C it became weaker contrasting with the higher number of patients. Since AUROCs for LSPS also did not exceed 0.8 at any of the two HVPG cut-off levels evaluated in our study, we can claim that LSPS was not superior to LS alone for prediction of HVPG at 16 and 20 mm Hg. However, we can neither confirm nor call in question the excellent power of LSPS to predict CSPH in patients with less advanced liver cirrhosis [40].

ELF has originally been constructed as a non-invasive predictor of liver fibrosis stage in non-cirrhotic patients with chronic liver disease [45, 55]. Since ELF represents a reliable tool for distinction between cirrhotic and non-cirrhotic patients, we would have expected a good correlation with HVPG. In reality, both ELF score and its individual components HA, PIIINP and TIMP-1, showed a weak correlation with HVPG in our cohort. Simbrunner et al. [56] published recently a study larger than ours showing better correlation between ELF and HVPG in their cohort of patients of Child-Pugh A, B and C. However, most of the patients (116/201, 58%) were in the Child-Pugh A group and the authors achieved a significant correlation between ELF and HVPG only in Child-Pugh A patients but not in Child-Pugh B and C patients. The authors also demonstrated that the strength of the correlation between ELF and HVPG decreased at higher HVPG levels. Owing to high proportion of Child-Pugh A class patients, they were able to predict CSPH accurately with AUROC 0.833 in the whole cohort. Consistently with the Simbrunner’s results, we achieved a weaker correlation in our group with predominance of CPS B/C patients, the correlation between ELF and HVPG in CPS A patients was better than in the entire cohort but weaker than in the Simbrunner’s study; in our CPS-B/C group, the correlation was marginally significant. This suggests that ELF score may indeed serve as a non-invasive predictor of HVPG with a better performance in Child-Pugh A patients.

Despite all efforts, our results cannot be extrapolated to general population of patients with advanced liver disease. Nonetheless, the data strongly suggest the feasibility of the non-invasive prediction of HVPG at higher cut-off points by LS.

HVPG is currently the best validated tool to assess prognosis of patients with liver cirrhosis. In this study, we demonstrated the strongest correlation between LS and HVPG in wide range of HVPG values, so far only in a specific group of patients without ongoing alcohol abuse and significant liver steatosis. We speculate that with further technical development in the field of non-invasive assessment of liver fibrosis and steatosis, LS measurement will be able to fully substitute for invasive HVPG measurement to evaluate the risks associated with advanced liver disease.

## Supporting information

S1 File Patients’ clinical and laboratory data. (XLSX)

## Abbreviations

2D-SWE: two-dimensional real time shear-wave elastography
AUROCs: areas under the receiver operating characteristic curves
CPS: Child-Pugh score
CSPH: clinically significant portal hypertension
HA: hyaluronic acid
HCC: Hepatocellular carcinoma
HVPG: Hepatic venous pressure gradient
IL-1ra/IL-1F3: Interleukin-1 Receptor Antagonist
IL-6: Interleukin-6
LR: likelihood ratios
LS: Liver stiffness
LSPS: Liver Spleen Platelets Score
MELD: Model for End-Stage Liver Disease
NPV: negative predictive value
NSBB: non-selective β-blockers
PH: Portal hypertension
PIIINP: Amino-Terminal Propeptide of Type III Procollagen
PP: portal pressure
PPV: positive predictive value
TIMP-1: Tissue Inhibitor of Matrix Metalloproteinase 1
TNFα: Tumour Necrosis Factor alpha
VCAM-1: Vascular Cell Adhesion Molecule 1
WHVP: wedge hepatic vein pressure
